# Efficiency of endoscopic artificial intelligence in the diagnosis of early esophageal cancer

**DOI:** 10.1111/1759-7714.15261

**Published:** 2024-04-29

**Authors:** Yongkang Tao, Long Fang, Geng Qin, Yingying Xu, Shuang Zhang, Xiangrong Zhang, Shiyu Du

**Affiliations:** ^1^ Department of Gastroenterology China‐Japan Friendship Hospital Beijing China; ^2^ Beijing University of Chinese Medicine Beijing China

**Keywords:** artificial intelligence, depth of infiltration, diagnosis, early esophageal cancer, endoscopy

## Abstract

**Background:**

The accuracy of artificial intelligence (AI) and experts in diagnosing early esophageal cancer (EC) and its infiltration depth was summarized and analyzed, thus identifying the advantages of AI over traditional manual diagnosis, with a view to more accurately assisting doctors in evaluating the patients' conditions and improving their cure and survival rates.

**Methods:**

The PubMed, EMBASE, Cochrane, Google, and CNKI databases were searched for relevant literature related to AI diagnosis of early EC and its invasion depth published before August 2023. Summary analysis of pooled sensitivity, specificity, summary receiver operating characteristics (SROC) and area under the curve (AUC) of AI in diagnosing early EC were performed, and Review Manager and Stata were adopted for data analysis.

**Results:**

A total of 19 studies were enrolled with a low to moderate total risk of bias. The pooled sensitivity of AI for diagnosing early EC was markedly higher than that of novices and comparable to that of endoscopists. Moreover, AI predicted early EC with markedly higher AUCs than novices and experts (0.93 vs. 0.74 vs. 0.89). In addition, pooled sensitivity and specificity in the diagnosis of invasion depth in early EC were higher than that of experts, with AUCs of 0.97 and 0.92, respectively.

**Conclusion:**

AI‐assistance can diagnose early EC and its infiltration depth more accurately, which can help in its early intervention and the customization of personalized treatment plans. Therefore, AI systems have great potential in the early diagnosis of EC.

## Graphical abstract

To identify the advantages of artificial intelligence (AI) over traditional manual diagnosis in diagnosing early esophageal cancer (EC), we searched the relevant literature up to August 2023 and performed a summary analysis. A total of 19 studies were enrolled with a low to moderate total risk of bias. The pooled sensitivity of AI for diagnosing early EC was markedly higher than that of novices and comparable to that of endoscopists. Moreover, AI predicted early EC with markedly higher AUCs than novices and experts. In addition, pooled sensitivity and specificity of AI in the diagnosis of invasion depth in early EC were higher than that of experts. Therefore, AI‐assistance can diagnose early EC and its infiltration depth more accurately. SROC curves for early EC diagnosis (a) and its invasion depth (b) by AI and traditional manual diagnosis.

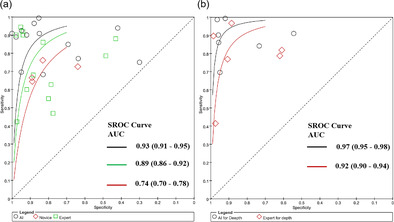

## INTRODUCTION

Esophageal cancer (EC) is a malignant tumor of the digestive tract with high incidence, and about 500 000 people suffer from EC every year worldwide.^1^ As the early symptoms of EC are not obvious, when the patients show relevant symptoms and go to the hospital for consultation, they often have already reached the middle or late stage and missed the best treatment period.^2^ According to statistics, once the disease develops into middle or late stage, the 5‐year survival rate of EC patients will drop from 75% to about 12%, which greatly reduces their quality of life.^3^ Recently, the incidence of EC has been increasing year by year due to poor dietary habits, such as high salt, high fat, fried food, long‐term smoking, heavy drinking, and HP infection, and there is a tendency of youthfulness. Therefore, early diagnosis of EC is increasingly important.^4^ Through early diagnosis, EC patients can receive less invasive treatments, thus reducing discomfort, decreasing complexity, and prolonging survival. It also has a positive impact on optimizing the allocation of healthcare resources and reducing patients' healthcare costs. Therefore, regular screening and seeking medical advice are crucial for individuals at high risk of EC or those with symptoms.

Currently, there are various EC screening modalities used in clinical work, such as endoscopy, imaging surveillance, tumor marker screening, and pathological biopsy.^5^ Among them, the importance of endoscopy should not be overlooked, as it provides crucial information by directly observing the tissues inside the esophagus, thus detecting the presence of EC at an early stage.^6^ Meanwhile, endoscopy can assist in collecting tissue samples for biological examination, and determine the type, grade and stage of the cancer, thus guiding the treatment plan.^7^ In addition, it helps monitor the effectiveness of treatment and recurrence of cancer, providing patients with better treatment and prognostic opportunities. Therefore, endoscopy is important in the overall management of EC. However, it is undeniable that the accuracy of endoscopic screening is inextricably linked to the experience of the surgeon. Shortcomings in endoscopic diagnosis of EC mainly include the inexperience of the surgeon, the difficulties in the operation, as well as the insidious nature of certain lesions, especially in the early stages of EC.^8^ Therefore, improving the skill level of physicians and the use of assistive technologies is essential for improving the early diagnosis of EC.

With the development of artificial intelligence (AI) technology, its application in the medical field is extensive and profound, including medical image analysis, drug discovery, genomics, remote monitoring, surgical assistance, and medical literature analysis, which plays a key role in improving the quality, efficiency, and personalization of healthcare.^9^ Currently, AI is also being used to assist endoscopy in the diagnosis of EC, with its ability to analyze endoscopic images in real time to be able to detect tiny abnormalities or lesions, thus helping doctors locate problem areas more quickly and accurately.^10^ In addition, AI systems can improve the efficiency of doctors, make more efficient use of healthcare resources, and improve their diagnostic capabilities over time to provide better healthcare services to patients.^11^ Therefore, AI represents great promise for use in healthcare.

However, it is undeniable that the application of AI systems relies on data quality, which may be affected by factors such as image quality and lighting, in addition to interpretability issues and the risk of false alarms and omissions in AI systems that may affect patient diagnosis and treatment. Therefore, in the present study we aimed to investigate the diagnostic efficacy of the AI system in early EC and its infiltration depth, thus better improving the treatment and survival chances of EC patients.

## METHODS

### Data retrieval and collection

A literature search was carried out using PubMed Embase, Cochrane, Google, and CNKI databases. The keywords used were: “early” or “superficial cancer”and “esophageal cancer” or “esophageal tumors” or “esophageal squamous cell carcinoma” or “Barrett's esophagus” or “infiltration depth” or “SM1”and “artificial intelligence” or “deep learning” or “convolutional neural network (CNN)” or “computer aided” or “manual diagnosis” and “sensitivity” or “specificity” or “expert” or “specialist” or “novice” or “young doctor” and “endoscope” or “narrow‐band imaging (NBI)” or “white‐light imaging (WLI)”. Relevant studies were searched from January 2000 to August 2023 by two researchers with 3 years of searching experience.

### Inclusion criteria

(1) Diagnose early EC or its infiltration depth. (2) Analysis of endoscopic images of early esophageal cancer or benign esophageal‐related lesions. (3) Diagnosis using AI or CNNs. (4) Include manual diagnosis by endoscopists or beginners as a control. (5) Include relevant diagnostic metrics, such as accuracy, sensitivity, and specificity. (6) Pathology report results as the gold standard.

### Exclusion criteria

(1) Case reports, reviews, and meta‐analysis. (2) Animal‐related research. (3) The number of cases included in the study was less than 10. (4) Unable to obtain complete data. (5) Lack of relevant study indicators.

### Data extraction

The authors, year of publication, endoscopic image types, AI model types, diagnostic methods, values of true positives (TP), false positives (FP), false negatives (FN), true negatives (TN) in diagnosing early EC or its infiltration depth were extracted from the literature. When necessary, we contacted the authors by email to acquire further details of the data.

### Quality evaluation of included literature

Quality Assessment of Diagnostic Accuracy Studies‐2 check list was adopted to evaluate the quality of literature.

### Statistical analysis

Revman 5.3 and Stata14 were used. The pooled sensitivity and specificity of AI‐assisted and manual diagnosis for early EC and its infiltration depth were calculated and forest plots were made. In addition, we further plotted the summary receiver operating characteristics (SROC) curve and calculated the area under the curve (AUC) to further predict the accuracy of the diagnosis. Furthermore, funnel plots were drawn to assess whether there was publication bias in the included studies, with *p* < 0.1 suggesting that the funnel plots were asymmetric.

## RESULTS

### Literature search

**FIGURE 1:**
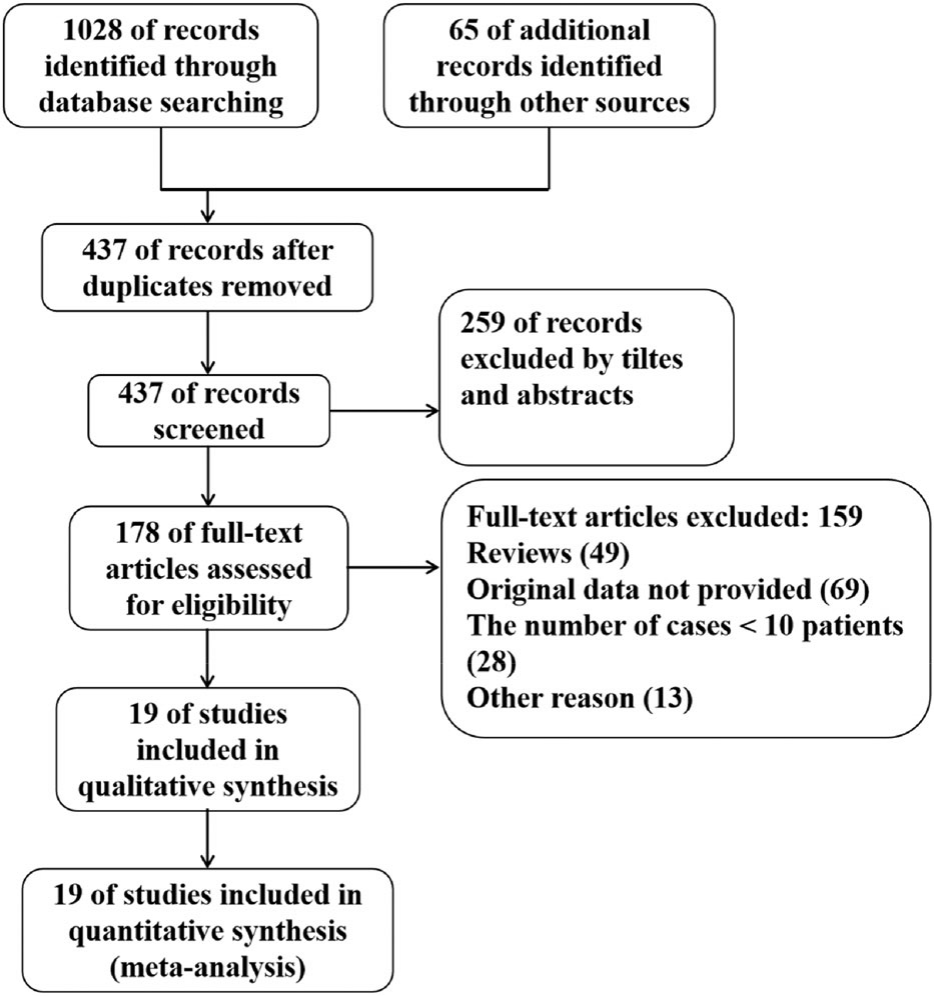
Flow chart for data search and gathering.

A total of 1093 publications on AI‐assisted diagnosis of early EC and its infiltration depth were searched, of which 656 were duplicates. Through initial screening, 259 studies were excluded because their titles and abstracts did not meet our requirements. Subsequently, after reading through the full text of 178 publications, 159 were further excluded (reviews [49], detailed data were not provided [69], the number of people included in the study was less than 10 [28], and meta‐analysis [13]). Finally, 19 papers relating to AI‐assisted diagnosis of early EC and its invasion depth were included in the study.^6^, ^7^, ^8^, ^9^, ^10^, ^11^, ^12^, ^13^, ^14^, ^15^, ^16^, ^17^, ^18^, ^19^, ^20^, ^21^, ^22^, ^23^, ^24^ By analyzing endoscopic WLI/NBI, the sensitivity and specificity of AI‐assisted systems, novices, and experts in the diagnosis of early EC and its infiltration depth were compared in these studies, respectively. The literature search process is shown in Figure 1, and the basic information of each study was extracted (Table 1).

**TABLE 1:** Basic information and relevant diagnostic indicators of included studies.

Authors and year	Imaging type	AI model	Diagnostic methods	Diagnosis of esophageal cancer				Diagnosis of invasive EC			
				TP	FP	FN	TN	TP	FP	FN	TN
Ebigbo et al. (2021)^6^	WLI	CNN	AI	47	20	14	35				
			Expert	37	12	22	44				
Everson et al. 2021^7^	NBI	CNN	AI					73	3	1	81
			Expert					119	4	4	31
Horie et al. 2019^8^	WLI + NBI	CNN	AI					142	2	1	23
Hussein et al. 2022^9^	WLI	CNN	AI	27	4	1	29				
			Expert	22	17	6	16				
Ikenoyama et al. 2021^10^	WLI + NBI	CNN	AI	28	12	5	27				
			Expert	15	9	17	31				
Iwagami et al. 2021^11^	WLI/NBI	CNN	AI	107	76	7	55				
			Expert	95	78	13	59				
Li et al. 2021^12^	WLI/NBI	CNN	AI	100	0	10	2				
			Novice	31	7	17	57				
			Expert	85	1	5	20				
Meng et al. 2022^13^	WLI + NBI	CNN	AI	219	5	18	91				
			Novice	169	17	53	84				
			Expert	216	5	24	78				
Nakagawa et al. 2019^14^	WLI + NBI	Single‐shot multibox detector	AI					695	6	76	137
			Expert					704	15	80	1115
Shimamoto et al. 2020^15^	WLI + NBI	CNN	AI	16	4	7	75	16	4	7	75
			Expert	11	2	15	74	10	2	14	76
Shiroma et al.^16^ 2021	WLI + NBI	CNN	AI	15	14	5	6				
			Expert	11	4	9	16				
Tani et al. 2023^17^	NBI	CNN	AI	43	54	20	263				
			Expert	42	25	28	285				
Tokai et al. 2020^18^	WLI + NBI	CNN	AI					159	24	30	66
			Expert					149	34	40	56
Uema et al. 2021^19^	NBI	CNN	AI					623	1	101	22
			Expert					533	5	159	50
Wang et al. 2022^20^	WLI/NBI	CNN	AI	8	2	1	90				
			Novice	12	9	6	74				
			Expert	9	2	1	89				
Yang et al. 2021^21^	WIL	CNN	AI	506	81	51	459				
			Novice	717	40	269	71				
			Expert	474	93	77	454				
Yao et al. 2023^22^	WLI	CNN	AI	158	5	1	29				
			Expert	102	5	48	38				
Yuan et al. 2022^23^	NBI	CNN	AI					2071	290	205	346
			Expert					1932	217	424	339
Zhao et al. 2022^24^	NBI	CNN	AI	45	4	5	46				
			Expert	46	3	4	47				

Abbreviations: AI, artificial intelligence; CNN, convolutional neural network; FN, false negatives; FP, false positives; NBI, narrow‐band imaging; TP, true positives; TN, true negatives; WLI, white‐light imaging.

### Quality evaluation

**FIGURE 2:**
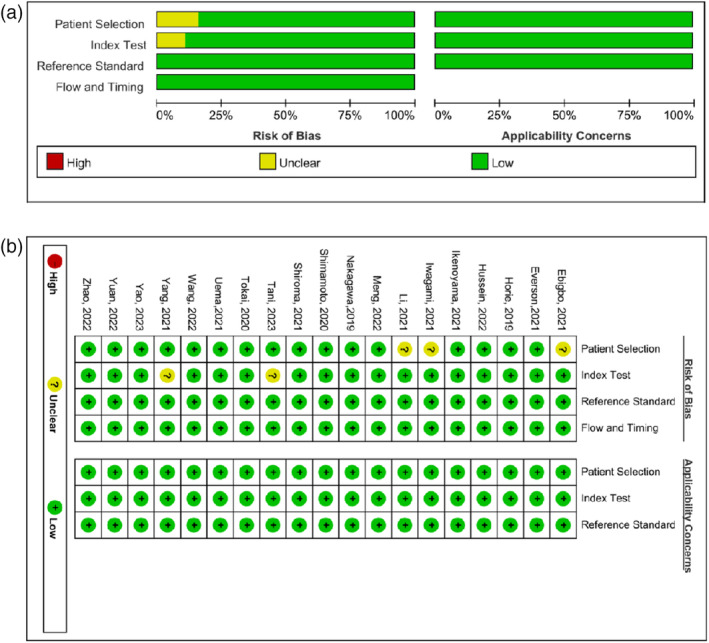
The risk of bias and applicability concerns graph (a) and summary (b) of included studies.

We found that the total risk of bias of the included literatures was low to moderate, and the risk of bias was unclear mainly in the “patient selection” and “index test”, with three and two studies, respectively. The rest were at low risk in all studies (Figure 2).

### AI‐assisted could more effectively improve the diagnosis of early EC

**FIGURE 3:**
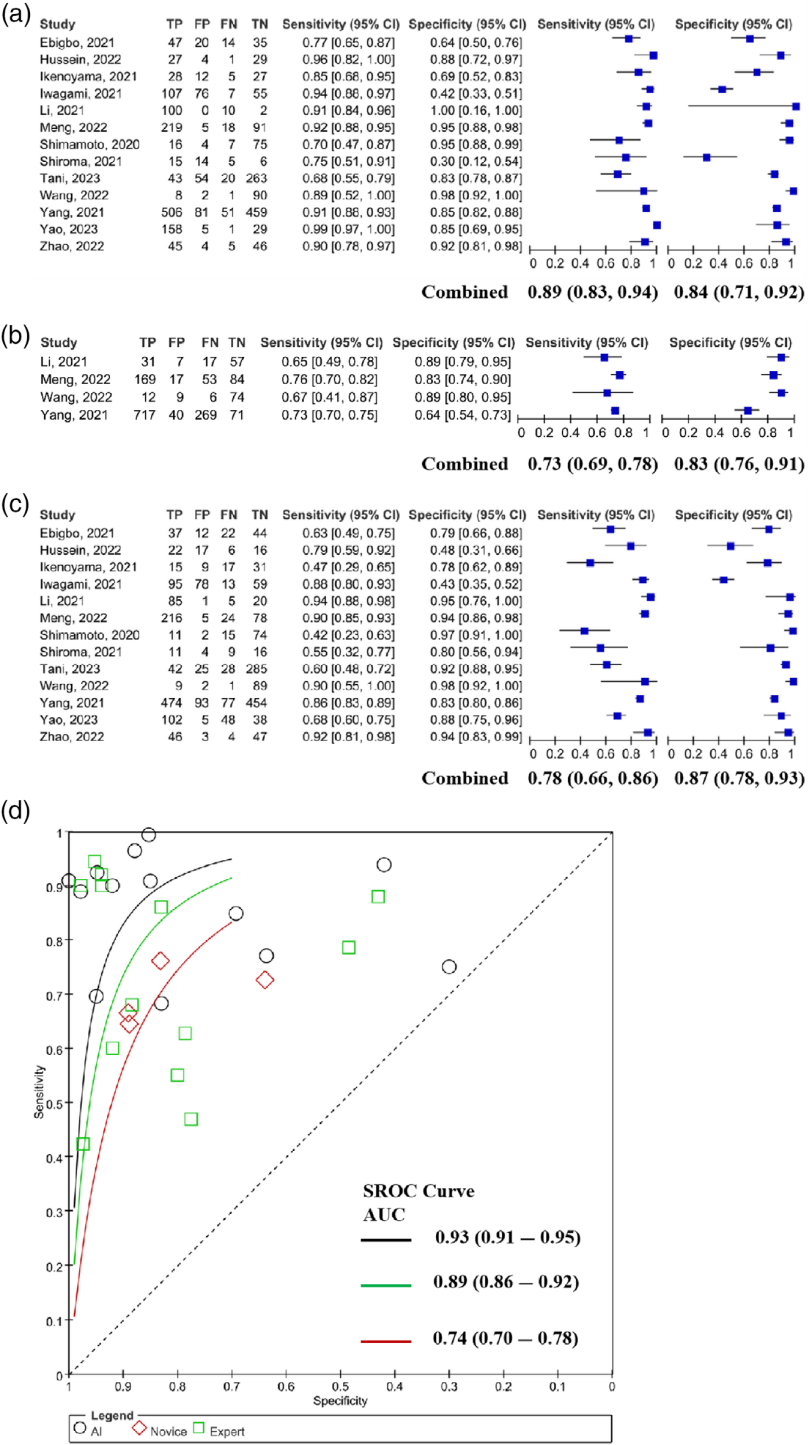
Analysis of the diagnosis of early esophageal cancer (EC). (a–c): Forest plots for artificial intelligence (AI), novices, and experts to diagnose early EC. (d): Summary receiver operating characteristics (SROC) curves for early EC diagnosis by AI, novices, and experts.

Improving the early diagnosis rate of EC and reducing missed diagnosis is an important guarantee for improving patients' prognosis. We summarized data from 13 papers related to early diagnosis of EC and explored the diagnostic efficacy of AI‐assisted diagnostic systems, novices, and experts, respectively. The results showed that the sensitivity of AI for diagnosing early EC was higher than that of novices and experts, and especially better than that of novices, with the pooled sensitivity values of 0.89 (95% confidence interval [CI]: 0.83–0.94), 0.73 (95% CI: 0.69–0.78), and 0.78 (95% CI: 0.66–0.86), and the pooled specificity values of 0.84 (95% CI: 0.71–0.92), 0.83 (95% CI: 0.76–0.91), and 0.87 (95% CI: 0.78–0.93) (Figure 3c). In addition, the SROC curves suggested that AI had higher diagnostic efficacy compared to novices and experts with pooled AUCs of 0.93, 0.74, and 0.89, respectively (Figure 3d). Therefore, AI‐assisted endoscopy can effectively reduce the rate of missed diagnosis during the diagnosis of early EC, which is an effective adjunct for both novices and experts.

### AI‐assisted can be more effective in assessing lesion depth in early EC

**FIGURE 4:**
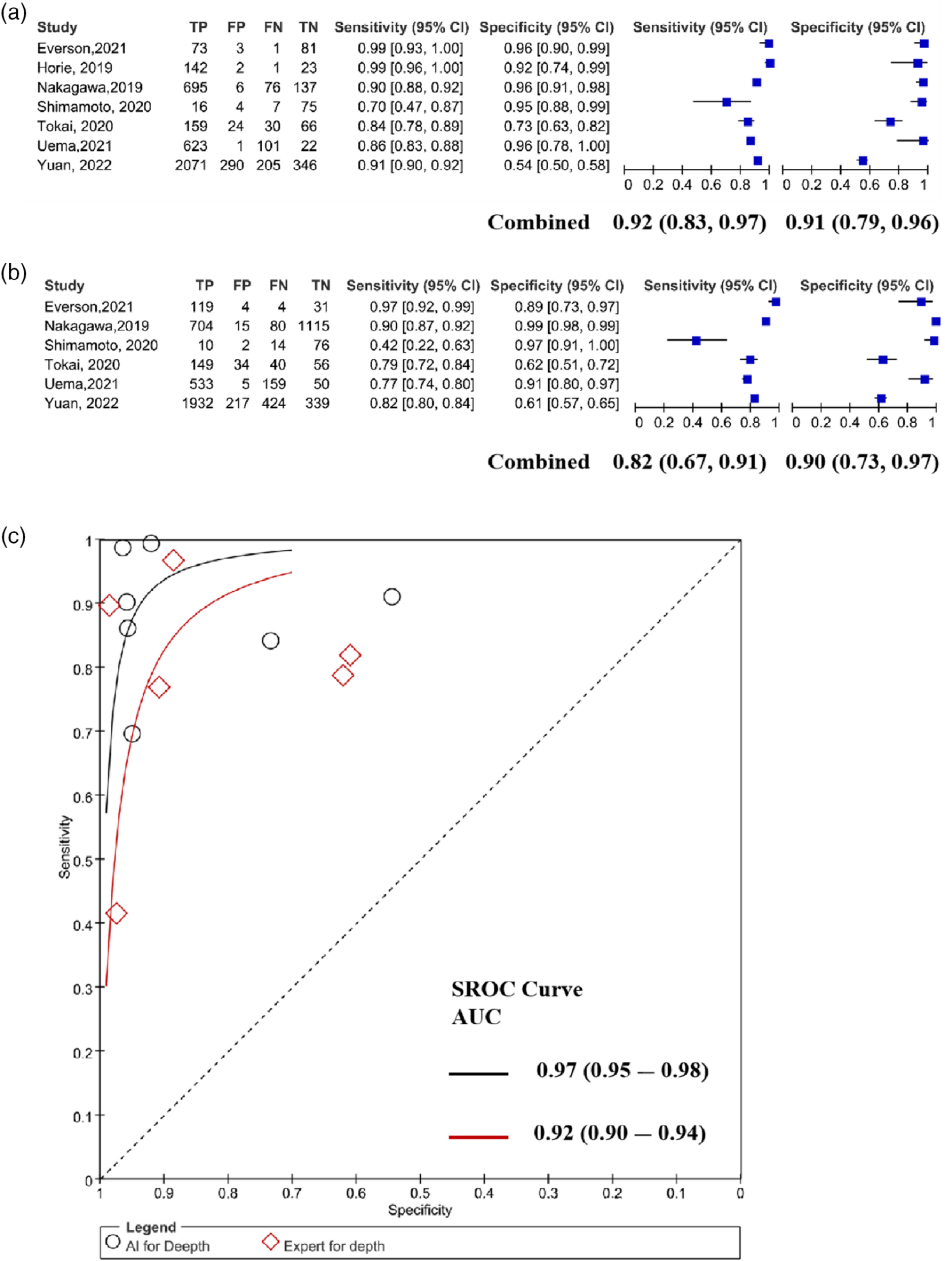
Analysis of the diagnosis of early esophageal cancer (EC') invasion depth. (a, b) Forest plots for artificial intelligence (AI) and experts to diagnose early invasion depth of EC. (c) Summary receiver operating characteristics (SROC) curves for early invasion depth of EC diagnosis by AI and experts.

In early‐stage EC diagnosis, the correct assessment of infiltration depth is a key factor in treatment decision‐making. We summarized the data related to the diagnosis rate of early EC infiltration depth in seven papers and investigated the diagnostic efficacy of AI and experts respectively. The results showed that the AI diagnosed early EC with higher sensitivity and specificity than experts, especially the sensitivity. The pooled values of sensitivity were 0.92 (95% CI: 0.83–0.97) and 0.82 (95% CI: 0.67–0.91), and the pooled specificity values were 0.91 (95% CI: 0.79–0.96) and 0.90 (95% CI: 0.73–0.97) (Figure 4a,b). In addition, the SROC curves suggested that AI had higher diagnostic efficacy for the invasion depth of early EC compared to experts, with pooled AUCs of 0.97 and 0.92, respectively (Figure 4c). Therefore, AI‐assisted endoscopy can reach the level of endoscopists in the diagnosis of infiltration depth of early EC.

### Publication bias analysis of included literature

**FIGURE 5:**
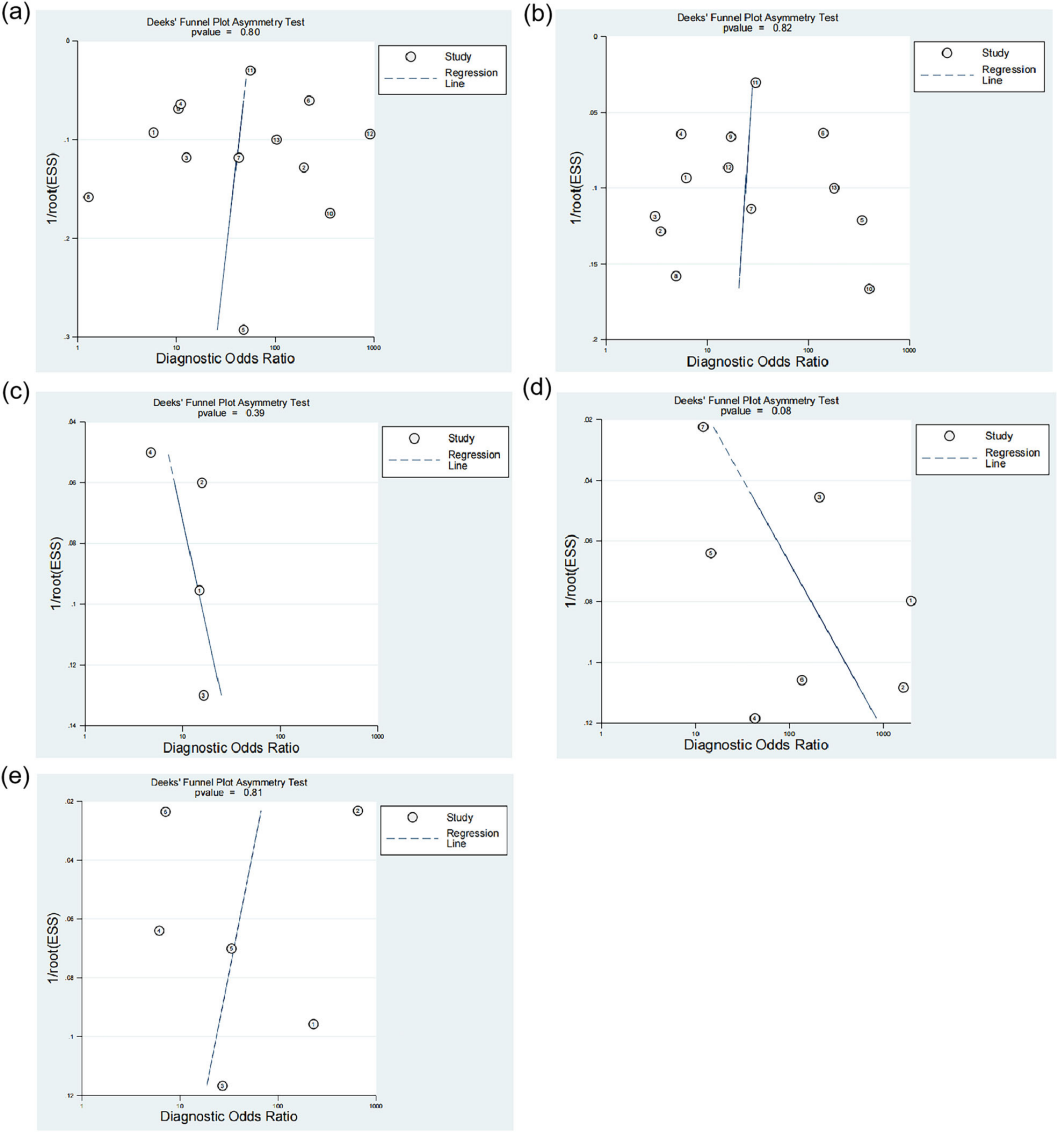
Publication bias analysis of included literature. (a–c) Funnel diagram of early esophageal cancer (EC) diagnosis by artificial intelligence (AI), novices and experts. (d, e) Funnel diagram of early EC' invasion depth by AI and experts.

We performed publication bias analyses for the sensitivity and specificity of AI, novices, and experts in diagnosing early EC and its infiltration depth and found that the funnel plots of the above outcomes were largely symmetrical (Figure 5), indicating no obvious publication bias.

## DISCUSSION

Screening and diagnosis of early EC through endoscopy facilitates medical personnel to detect lesions under direct vision and perform relevant biopsies and surgical treatments. However, since mucosal lesions of early EC are not obvious and not easily detected by the naked eye, it requires endoscopists to accumulate rich clinical working experience and professional knowledge for identifying and locating these tiny lesions in time to make accurate diagnosis.^12^ Identifying early, microscopic EC lesions can be challenging for young or inexperienced doctors, which may lead to missed diagnosis and delay patients' treatment.^13^ Moreover, endoscopy results may be affected by doctors' subjective judgment, which may lead to inconsistent diagnostic results between different doctors, thus leading to errors and affecting the diagnostic accuracy of early EC.^15^ Therefore, AI‐assisted systems have been introduced to aid endoscopists in the diagnosis of early EC, particularly in the identification of microscopic lesions. However, the results of studies on the diagnostic efficiency of AI systems in early EC have been reported inconsistently due to uncertainties in data quality, interpretability of results, and false positives and omissions.^18^, ^19^, ^20^ Therefore, we summarized and analyzed the sensitivity and specificity of the AI system for the diagnosis of early EC and its invasion depth in various studies, hoping to provide the best diagnostic support for patients.

In the comparison between AI‐assisted and manual diagnosis, AI has a clear advantage in the diagnosis of early EC, especially in relation to novices. The reason maybe that the diagnosis of early EC by doctors usually requires long‐term clinical experience and the accumulation of sufficient professional knowledge, and the insufficient reserve of young physicians in this area will lead to the increase of missed diagnosis. In contrast, AI‐assisted systems can be trained and learnt from large‐scale datasets, accumulating a wealth of cases and experience. This enables the AI system to identify ECs of different types and degrees of condition, including early lesions, thus improving the comprehensiveness of diagnosis.^17^ In addition, the AI system has a unique advantage in image analysis, which allows it to quickly and accurately analyze a large number of endoscopic images and effectively detect tiny lesions or abnormalities, thus helping in the timely detection of early EC. Yang et al.^21^ found that the AI system, trained through simulation, diagnosed early EC with an accuracy of 88.1% in the evaluation of 1097 WLI, which was higher than that of experienced endoscopists (84.5%) and low‐ranking physicians (68.5%), and there was an obvious increase in sensitivity (90.1% vs. 86.4% vs. 72.7%) and specificity (85% vs. 82.5% vs. 63.7%), showing that the AI system can reach the level of endoscopists after training. However, data from some studies have shown a certain number of false positives in AI diagnosis, thus resulting in over‐treatment. False positives were mainly found in endoscopic images regarding normal anatomy of the esophagogastric junction and cone compression, as well as benign lesions such as Barrett's esophagus, inflammation, and mucosal atrophy.^24^ Therefore, it is important to train the AI system to learn more about images of tiny lesions to better reduce the false positive rate.

The AI system also has an obvious advantage over manual diagnosis in the diagnosis of the infiltration depth of EC. The reason maybe that the AI system can effectively accumulate rich experience in the diagnosis of different types of EC and infiltration depth by processing endoscopic image data on a large scale.^16^ Meanwhile, the system analyses of AI are based on data and algorithms that are not affected by factors such as subjectivity, visual fatigue, or emotional fluctuations, and can provide consistent diagnostic results.^22^ More importantly, in the process of analyzing image data, the AI system can detect changes in subtle structures with a high degree of accuracy, thus improving the accuracy of infiltration depth diagnosis.^23^ Nakagawa^14^ et al. found that the accuracy (91% vs. 89.6%), sensitivity (90.1% vs. 89.8%), and specificity (95.8 vs. 88.3) of AI for the diagnosis of early ECs with infiltration depths above SM1 were differently improved than those of endoscopic specialists in the 914 endoscopic drawings. Therefore, AI‐assisted systems can provide high‐precision, high‐efficiency, and consistent diagnostic support in the diagnosis of early EC' infiltration depth.

There were certain shortcomings in this study. First, a comparison of the diagnostic efficiency of the AI‐assisted system with that of conventional endoscopic diagnostic methods was not performed, and therefore diagnostic times for AI‐assisted versus manual diagnosis were not compared, so evidence is lacking to demonstrate whether AI actually improves the diagnostic efficiency of early EC. Second, the diagnostic efficiency of AI‐assisted novice and experienced specialists was not compared, which is a key factor, as younger physicians may need AI support more. In follow‐up studies, further detailed data should be collected and summarized to more comprehensively and reliably assess the potential of AI‐assisted systems in the diagnosis of early EC.

In conclusion, trained with large‐scale data, the AI system demonstrated high accuracy and sensitivity for early EC, and its diagnostic efficacy was markedly higher than that of novices. Compared to endoscopists, the AI system performed at a comparable level and achieved appropriate improvements in some areas. Therefore, AI systems have great potential in the early diagnosis of EC and can become a powerful auxiliary tool in the medical field.

## AUTHOR CONTRIBUTIONS

Yongkang Tao contributed to research design, data collection, and analysis, and manuscript drafting; Long Fang contributed to study supervision and consultation; Geng Qin, Yingying Xu, Shuang Zhang, Xiangrong Zhang contributed to data collection, interpretation, and analysis; Shiyu Du contributed to study design, data analysis, and critical revision of the manuscript; all authors approved the final version and agree to be accountable for all aspects of the work.

## CONFLICT OF INTEREST STATEMENT

The authors declare no conflicts of interest.
